# Personality Functioning and Self-Disorders in different stages of Psychotic Disorders and Borderline Personality Disorder

**DOI:** 10.1192/j.eurpsy.2023.965

**Published:** 2023-07-19

**Authors:** M. Gruber, J. Alexopoulos, K. Feichtinger, K. Parth, A. Wininger, N. Mossaheb, F. Friedrich, Z. Litvan, B. Hinterbuchinger, S. Doering, V. Blüml

**Affiliations:** 1 Department of Psychoanalysis and Psychotherapy; Departement of Psychiatry and Psychotherapy, Division of Social Psychiatry; 2 Department of Psychoanalysis and Psychotherapy;; 3 Departement of Psychiatry and Psychotherapy, Division of Social Psychiatry, Medical University of Vienna, Vienna, Austria

## Abstract

**Introduction:**

Personality functioning, self-disorders and their relationship to psychotic symptoms on a continuum from mild attenuated experiences to manifest psychotic symptoms in psychotic disorders are highly relevant for psychopathology, course of illness and treatment planning in psychotic disorders, but empirical data is sparse.

**Objectives:**

This study aims at exploring personality functioning and self-disorders in individuals at ultra-high risk for psychosis (UHR) and with first-episode psychosis (FEP), compared to a clinical control group of subjects with borderline personality disorder (BPD) and healthy controls (HC).

**Methods:**

Personality functioning was measured in 107 participants (24 UHR, 29 FEP, and 27 BPD and 27 HC) using the Structured Interview for Personality Organization (STIPO) and the Level of Personality Functioning Scale (LPFS), and self-disorders were assessed using the Examination of Anomalous Self-Experience (EASE). A hierarchical cluster analysis was performed based on the seven STIPO dimensions.

**Results:**

Significant impairment in personality functioning was found in UHR (M = 4.29, SD = .908), FEP (M = 4.83, SD = 1.002), and BPD individuals (M=4.70, SD=.542) compared with HC (M = 1.63, SD = .565). FEP patients showed significantly worse overall personality functioning compared to UHR patients (p = .037). Patients with manifest psychosis (FEP) also exhibited significantly higher levels of self-disorders compared to BPD patients (p = .019). Self-disturbances in patients with milder forms of psychotic symptoms (UHR) were intermediate between the other diagnostic groups (FEP and BPD). Regardless of the main diagnoses, the three clusters of patients were found to differ in levels of personality functioning and self-disorder.

**Conclusions:**

Impairment of personality functioning varies in different stages of psychotic disorders. The level of self-disorders may allow differentiation between manifest psychosis and borderline personality disorder. An in-depth assessment of personality functioning and self-disorders could be helpful in differentiating diagnoses, treatment planning, and establishing foci for psychotherapeutic treatment modalities.

**Disclosure of Interest:**

M. Gruber: None Declared, J. Alexopoulos: None Declared, K. Feichtinger: None Declared, K. Parth: None Declared, A. Wininger: None Declared, N. Mossaheb: None Declared, F. Friedrich: None Declared, Z. Litvan: None Declared, B. Hinterbuchinger: None Declared, S. Doering: None Declared, V. Blüml Grant / Research support from: Grant / Research support from: Heigl-Foundation, Köhler-Foundation, International Psychoanalytical Association (IPA)

